# Longitudinal association of subjective sleep quality and health-related quality of life improvements with Vagus nerve stimulation in drug-resistant epilepsy: a 24-month retrospective cohort study

**DOI:** 10.3389/fsurg.2026.1914334

**Published:** 2026-08-18

**Authors:** Jiabo Xiao, Mengsi Ren, Bo Zhang, Shichang Guo, Xiaohui Liu, Binhong Li, Changzheng Dong

**Affiliations:** 1 Department of Neurosurgery II, Epilepsy Center, Hebei General Hospital, Shijiazhuang, China; 2 Department of Postgraduate, Hebei Medical University, Shijiazhuang, China; 3 Department of Neurology, The Second Hospital of Hebei Medical University, Shijiazhuang, China; 4 Department of Postgraduate, Hebei North University, Zhangjiakou, China

**Keywords:** drug-resistant epilepsy, PSQI, QOLIE-31, retrospective cohort study, vagus nerve stimulation

## Abstract

**Objective:**

To evaluate the longitudinal trajectories of subjective sleep quality and health-related quality of life (HRQOL) over a 24-month period in adult patients with drug-resistant epilepsy (DRE) following vagus nerve stimulation (VNS) implantation, and to explore the adjusted longitudinal association of these improvements alongside concurrent seizure-frequency reduction.

**Methods:**

This retrospective cohort study consecutively enrolled 29 adult patients (≥18 years) with DRE who underwent VNS implantation. Data regarding seizure frequency, Pittsburgh Sleep Quality Index (PSQI), and Quality of Life in Epilepsy-31 (QOLIE-31) scores were extracted from medical records at baseline, 6, 12, and 24 months post-implantation. Repeated-measures analysis of variance (RM-ANOVA) and generalized estimating equations (GEE) were utilized to assess temporal trends and adjust for concurrent seizure frequency (as a continuous, time-varying covariate) and baseline psychiatric comorbidities.

**Results:**

Over the 24-month follow-up, the cohort demonstrated a significant and progressive reduction in mean monthly seizure frequency. Concurrently, profound and sustained improvements were observed in both overall PSQI scores and total QOLIE-31 scores (all *p* < 0.001). Crucially, the re-analyzed GEE model revealed that the temporal improvements in sleep and HRQOL demonstrated a significant adjusted longitudinal association with VNS therapy. The time factor remained a highly significant predictor for improvements in both PSQI and QOLIE-31 (*p* < 0.01 for all time points) even when continuously adjusting for concurrent seizure frequency and baseline psychiatric comorbidities.

**Conclusion:**

VNS therapy provides significant, progressive, and sustained improvements in subjective sleep quality and overall HRQOL in adult patients with DRE. Furthermore, these neurobehavioral benefits demonstrate a robust adjusted longitudinal association that is not exclusively tethered to the degree of seizure-frequency reduction. However, causality and complete physiological independence cannot be established due to the observational, uncontrolled design.

## 1 Introduction

Drug-resistant epilepsy (DRE) affects approximately one-third of all patients with epilepsy and is associated with significant morbidity, heightened mortality risk, and severely impaired health-related quality of life (HRQOL) (1–3). For patients who are not candidates for resective surgery, vagus nerve stimulation (VNS) has become a cornerstone adjunctive neuromodulatory therapy (4, 5).

While the primary clinical objective of VNS is the attenuation of seizure frequency and severity, emerging literature increasingly emphasizes the secondary, yet equally critical, benefits of VNS (6). Among these, the interplay between sleep quality and epilepsy represents a bidirectional physiological relationship. Frequent seizures disrupt subjective sleep quality, and conversely, sleep deprivation is a well-documented seizure precipitant (7, 8). Patients with DRE frequently report chronic insomnia, daytime somnolence, and poor sleep efficiency, which profoundly degrade their daytime functioning and QOL (9, 10).

Although short-term studies hint at the mood-elevating and sleep-modulating effects of VNS (11), there remains a paucity of longitudinal data tracing the long-term (≥24 months) trajectories of sleep quality and QOL alongside seizure reduction (12). Furthermore, it is critical to determine whether improvements in QOL and sleep simply mirror seizure reduction or whether there is an adjusted longitudinal association indicating broader neuromodulatory effects that are not fully captured by seizure frequency counts.

Therefore, the objective of this 24-month retrospective cohort study is to rigorously quantify the temporal trajectories of subjective sleep quality (via PSQI) and HRQOL (via QOLIE-31) in patients with DRE treated with VNS, utilizing continuous time-varying covariates to adjust for concurrent seizure-frequency reduction, and to examine whether these temporal benefits exhibit an association with VNS therapy that is not solely dependent on the magnitude of seizure control.

## 2 Methods

### 2.1 Study population and ethical approval

This retrospective cohort study evaluated consecutive adult patients (age ≥18 years) diagnosed with drug-resistant epilepsy (DRE) according to the International League Against Epilepsy (ILAE) criteria, who underwent VNS implantation at the Epilepsy Center, Department of Neurosurgery, Hebei General Hospital between January 2018 and December 2023. Follow-up extended through June 2026.

**Inclusion Criteria:** (1) Adult patients aged ≥18 years at the time of surgery; (2) Confirmed diagnosis of DRE based on ILAE guidelines; (3) Underwent primary VNS implantation; (4) Completion of a minimum 24-month continuous post-implantation follow-up.

**Exclusion Criteria:** (1) Previous functional neurosurgery; (2) Progressive intracranial space-occupying lesions; (3) Severe cognitive impairment precluding the completion of psychometric questionnaires.

**Figure 1:**
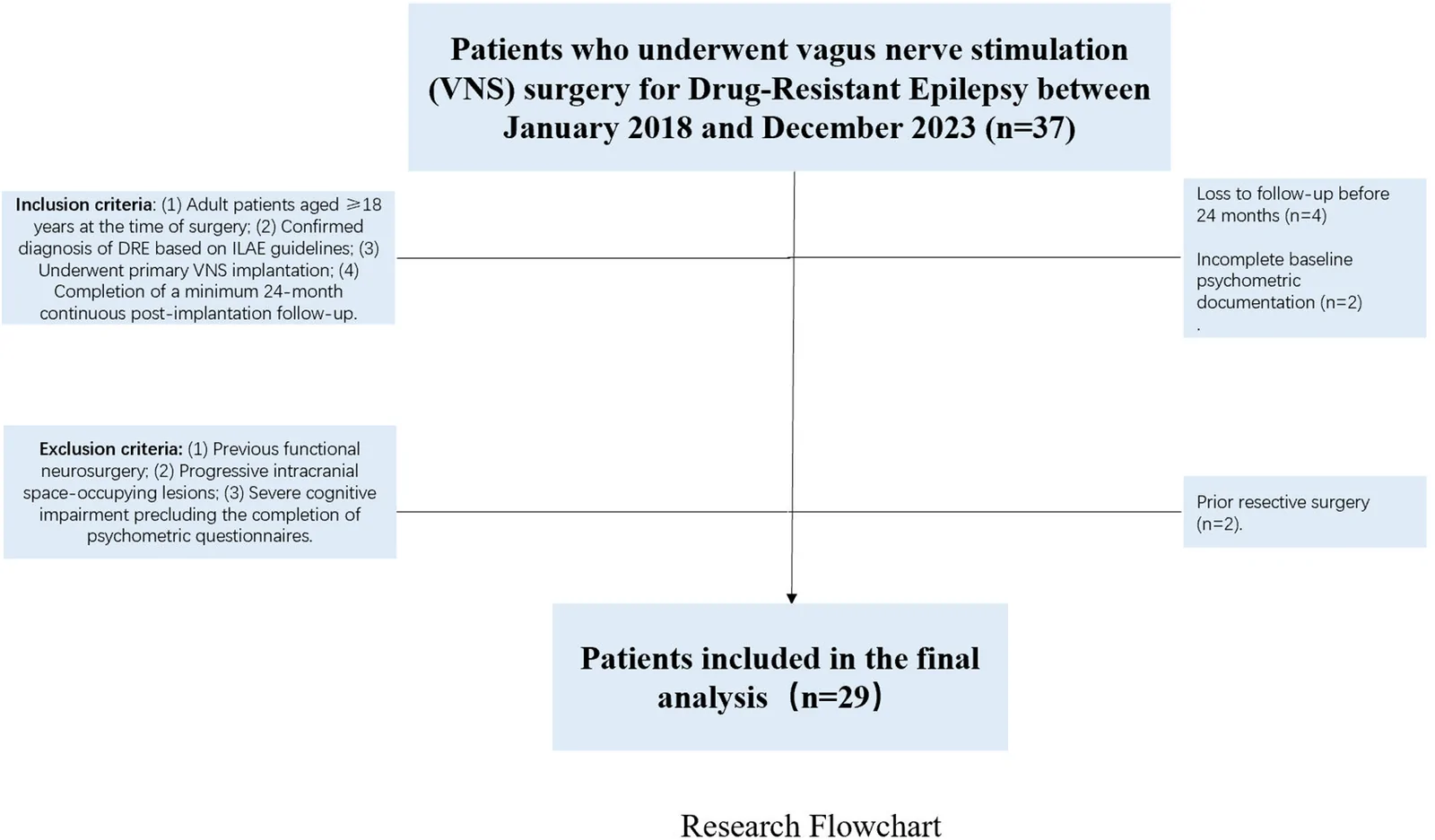
Research flowchart.

Following a rigorous clinical screening process of 37 initially identified patients, 8 patients were excluded from the final analysis. (4 due to loss to follow-up before 24 months, 2 due to incomplete baseline psychometric documentation, and 2 who underwent prior resective surgery). See Figure 1. Notably, the baseline psychometric assessments were conducted at a median interval of 7 days (range: 5–8 days) prior to the VNS implantation surgery.

The study protocol received formal ethical approval from the Ethics Committee of Hebei General Hospital (Approval No. 2025-LW-0125). Written informed consent was obtained from all participants or their designated legal representatives prior to surgical intervention, in strict adherence to the ethical principles outlined in the Declaration of Helsinki.

### 2.2 Surgical intervention and stimulation parameters

Patients were deemed eligible for neuromodulation following comprehensive preoperative evaluations—including continuous video-EEG monitoring—and multidisciplinary case discussions within the Epilepsy Center (13). All surgical procedures were performed by an experienced neurosurgical team utilizing a standardized operative protocol (14). Patients were implanted with the PINS VNS system (Model G112, PINS Medical Co., Ltd., Beijing, China), with the helical stimulation electrodes meticulously positioned and secured around the left cervical vagus nerve.

Postoperatively, device programming was typically initiated at a low current threshold (e.g., 0.25 mA) and progressively up-titrated. Standard stimulation parameters generally involved a pulse width of 250–500 µs and a frequency of 20–30 Hz, utilizing a duty cycle of 30 s of stimulation followed by 5 min of rest. All patients received standard electrical stimulation paradigms. Detailed stimulation parameters are provided in Supplementary Table S1.

### 2.3 Assessment tools and follow-up time points

Clinical efficacy and neurobehavioral parameters were systematically evaluated at baseline (pre-implantation) and at designated post-implantation follow-up points: 6, 12, and 24 months. Data acquisition relied on comprehensive medical record reviews, patient-maintained seizure diaries, and standardized psychometric questionnaires administered during routine outpatient visits. The longitudinal evaluation framework encompassed the following core domains:Seizure Outcomes: Quantified as the average monthly seizure frequency. Subjective sleep quality Evaluation: Subjective sleep quality and disturbances were assessed using the Pittsburgh Sleep Quality Index (PSQI) (15, 16). This self-rated instrument evaluates sleep patterns over a one-month interval, with a global score of ≥5 formally denoting a “poor” sleeper (17). Health-Related Quality of Life (HRQOL): Overall quality of life was measured utilizing the Quality of Life in Epilepsy-31 (QOLIE-31) inventory (18–20). This validated, disease-specific survey generates a scale from 0 to 100, where higher aggregate scores directly reflect superior health-related quality of life and psychosocial well-being.

### 2.4 Statistical analysis

All analyses were performed using SPSS software (version 27.0; IBM Corporation, Armonk, NY, USA) and R software (version 4.5.1; Statistical Computing R Foundation, Vienna, Austria). Continuous variables are expressed as mean ± standard deviation or median (interquartile range), while categorical data are presented as counts and percentages. To analyze the temporal trajectories of seizure frequency, PSQI, and QOLIE-31 scores, we utilized Repeated-Measures Analysis of Variance (RM-ANOVA) to detect changes over the four time points. To account for the inherent intra-subject correlation of longitudinal data, a Generalized Estimating Equations (GEE) model was implemented. Statistical significance was set at a two-tailed *p* < 0.05.

## 3 Results

### 3.1 Baseline data and clinical characteristics

This study included a total of 29 adult patients diagnosed with drug-resistant epilepsy (DRE) who underwent VNS implantation. The cohort comprised 12 males (41.4%) and 17 females (58.6%), with a mean age at VNS implantation of 36.6 ± 11.9 years. The average duration of epilepsy prior to neuromodulation was 14.7 ± 5.7 years. At baseline, the median seizure frequency was 20.0 (interquartile range IQR: 11.0–25.0) per month, and patients were concurrently taking an average of 3.7 ± 1.0 antiseizure medications (ASMs).

Baseline evaluations of sleep and quality of life demonstrated significant pre-operative impairments. The mean baseline Pittsburgh Sleep Quality Index (PSQI) score for the cohort was 8.2 ± 3.4, with 24 patients (82.8%) categorized as "poor" sleepers (PSQI score ≥5). The baseline overall Quality of Life in Epilepsy-31 (QOLIE-31) score was 48.5 ± 13.2. Detailed baseline demographic and clinical variables are summarized in Table 1.

**Table 1:** Baseline demographic and clinical characteristics of patients with DRE (*N* = 29).

Characteristic	Total Cohort（*N* = 29)
Demographics	
Sex, male (*n*, %)	12 (41.4%)
Age at VNS, years (Mean ± SD)	36.6 ± 11.9
Epilepsy History	
Age at seizure onset, years (Mean ± SD)	21.9 ± 10.4
Duration of epilepsy, years (Mean ± SD)	14.7 ± 5.7
Clinical Seizure Baseline	
Number of ASMs at baseline (Mean ± SD)	3.7 ± 1.0
Seizure frequency at baseline (per month)	19.0 ± 14.2
Epilepsy Etiology	
Structural (*n*, %)	4（13.8%）
Unknown/Cryptogenic (*n*, %)	25（86.2%）
Seizure Types	
Focal impaired awareness seizures (*n*, %)	15 (51.7%)
Focal to bilateral tonic-clonic seizures (*n*, %)	10 (34.5%)
Generalized onset seizures (*n*, %)	4 (13.8%)
Psychiatric Comorbidities & Psychometric Assessments	
Baseline HAMD score (Mean ± SD)	8.7 ± 3.2
Baseline HAMA score (Mean ± SD)	9.1 ± 4.5
Presence of Psychiatric Comorbidity, *n* (%)	27（93.1%）
Diagnosed Sleep Apnea, *n* (%)	2 (6.9%)
Sedative-Hypnotic Drug Use, *n* (%)	7 (24.1%)
Baseline PSQI score (Mean ± SD)	8.2 ± 3.4
Baseline QOLIE-31 score (Mean ± SD)	48.5 ± 13.2

ASM, antiseizure medication; DRE, drug-resistant epilepsy; HAMA, Hamilton Anxiety Rating Scale; HAMD, Hamilton Depression Rating Scale; PSQI, Pittsburgh Sleep Quality Index; QOLIE-31, Quality of Life in Epilepsy-31; SD, standard deviation; VNS, vagus nerve stimulation. (Psychiatric comorbidity is defined as HAMD or HAMA score ≥8).

### 3.2 Longitudinal changes in seizure frequency, sleep quality, and HRQOL

#### 3.2.1 Trajectory of seizure frequency reduction

**Figure 2:**
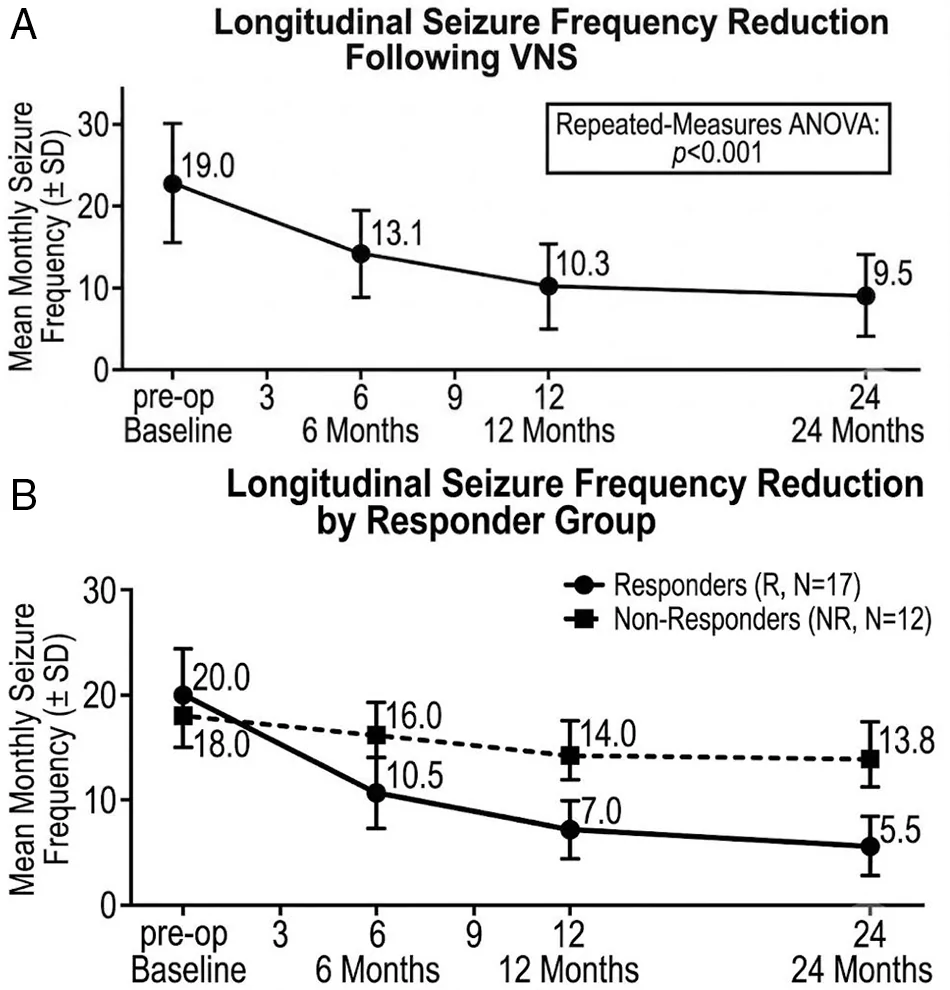
Longitudinal reduction in seizure frequency following vagus nerve stimulation (VNS) implantation. **(A)** The line graph illustrates a significant and progressive decrease in the mean monthly seizure frequency over the 24-month follow-up period among the total cohort of 29 patients with drug-resistant epilepsy (DRE). **(B)** Longitudinal seizure frequency reduction stratified by VNS response status, comparing Responders (R, *N* = 17) and Non-Responders (NR, *N* = 12). Data are presented as mean ± standard deviation (SD). Repeated-measures analysis of variance (RM-ANOVA) confirmed a highly significant main effect of time on the reduction of seizure frequency (*p* < 0.001).

Following VNS implantation, the cohort demonstrated a significant and progressive reduction in mean monthly seizure frequency over the 24-month observation period (Table 2). The mean seizure frequency decreased from a baseline of 19.0 ± 14.2 to 13.1 ± 11.5 at 6 months, 10.3 ± 8.8 at 12 months, and 9.5 ± 8.6 at 24 months. Repeated-measures ANOVA (RM-ANOVA) confirmed a highly significant main effect of time on seizure reduction (*p* < 0.001) (Figure 2A). When stratified by VNS response status, both Responders (R, *N* = 17) and Non-Responders (NR, *N* = 12) exhibited distinct longitudinal trajectories in their seizure frequency reduction over the 24-month period (Figure 2B).

**Table 2:** Longitudinal changes in seizure frequency, sleep quality, and health-related quality of life over the 24-month follow-up period.

Clinical Measure	Baseline (*N* = 29)	6 Months (*N* = 29)	12 Months (*N* = 29)	24 Months (*N* = 29)	*p*-value
Seizure Frequency (per month)	19.0 ± 14.2	13.1 ± 11.5	10.3 ± 8.8	9.5 ± 8.6	<0.001*
PSQI Total Score	8.2 ± 3.4	6.8 ± 3.1	5.9 ± 2.8	5.5 ± 2.5	<0.001*
QOLIE-31 Total Score	48.5 ± 13.2	54.2 ± 14.1	58.6 ± 15.0	61.3 ± 14.8	<0.001*

Data are expressed as mean ± standard deviation (SD). *p*-values were calculated using Repeated-Measures ANOVA to assess the main effect of time across the four designated time points. Denotes statistical significance (*p* < 0.05).PSQI, Pittsburgh Sleep Quality Index; QOLIE-31, Quality of Life in Epilepsy-31.

The asterisk (*) denotes statistical significance at *p* < 0.05.

#### 3.2.2 Progressive improvements in subjective sleep quality (PSQI)

**Figure 3:**
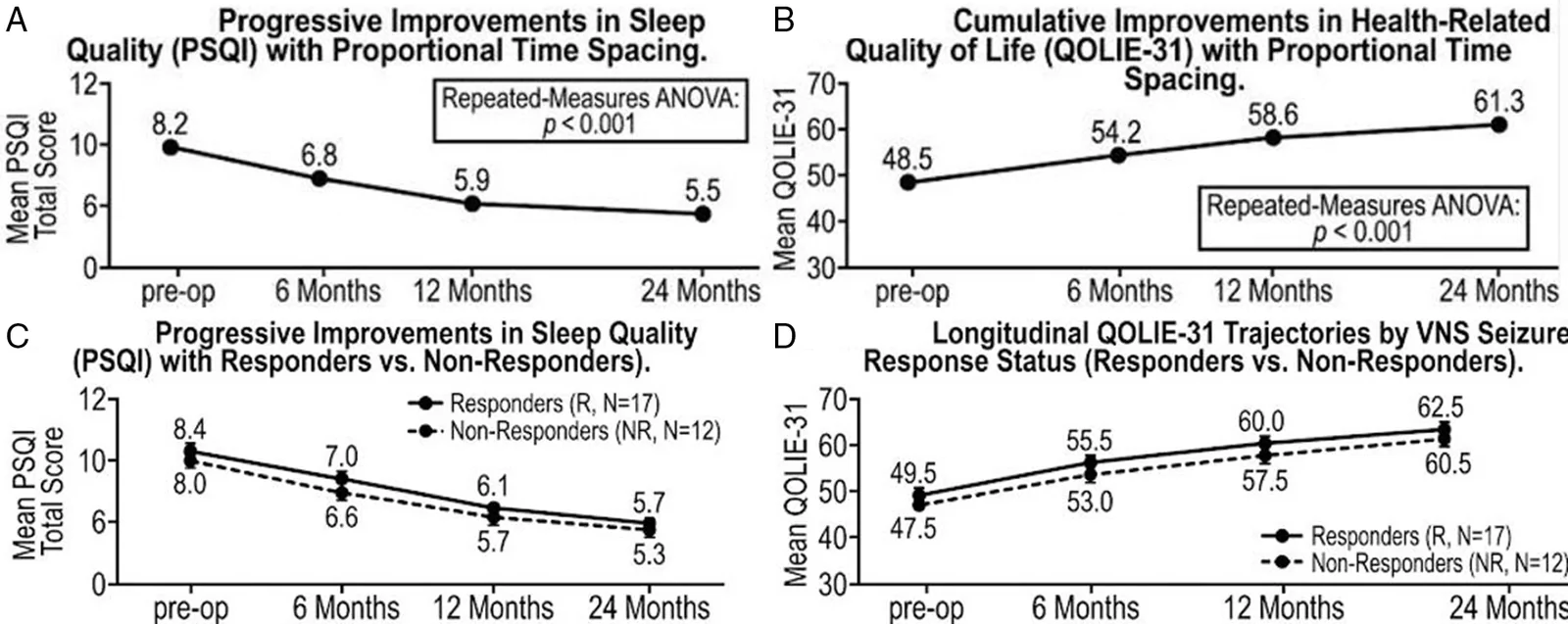
Progressive improvements in sleep quality and health-related quality of life (HRQOL) over 24 months. **(A)** The total Pittsburgh Sleep Quality Index (PSQI) score exhibited a significant longitudinal reduction, indicating profound and sustained improvements in subjective sleep quality across the overall cohort. **(B)** The total Quality of Life in Epilepsy-31 (QOLIE-31) score demonstrated a robust upward trajectory, reflecting steady, cumulative enhancements in overall HRQOL. **(C)** Longitudinal trajectories of PSQI scores stratified by VNS response status [Responders [R, *N* = 17] vs. Non-Responders [NR, *N* = 12]]. **(D)** Longitudinal QOLIE-31 trajectories stratified by VNS seizure response status. Panels C and D highlight that improvements in the longitudinal trajectories of sleep quality and QoL are similar across both groups, demonstrating that these benefits act independently of the strict seizure-reduction benefit. Both overall clinical measures showed a highly significant main effect of time across the designated follow-up points via RM-ANOVA (*p* < 0.001).

Subjective sleep quality, quantified by the PSQI, exhibited profound and sustained improvements across the entire cohort. The mean PSQI score decreased (indicating improved sleep quality) from a baseline of 8.2 ± 3.4–6.8 ± 3.1 at 6 months, reaching 5.9 ± 2.8 by 12 months, and 5.5 ± 2.5 at 24 months. RM-ANOVA demonstrated a significant longitudinal reduction in overall PSQI scores over the follow-up period (*p* < 0.001) (Figure 3A). Notably, when providing longitudinal trajectories by VNS response, these progressive improvements in sleep quality were remarkably similar between the Responder and Non-Responder groups (Figure 3C), indicating a benefit that is independent of the strict seizure-reduction benefit.

#### 3.2.3 Evolution of health-related quality of life (QOLIE-31)

Parallel to the sleep quality enhancements, the overall health-related quality of life (HRQOL) underwent steady, cumulative improvements. The mean total QOLIE-31 score increased from a baseline of 48.5 ± 13.2–54.2 ± 14.1 at 6 months, 58.6 ± 15.0 at 12 months, and peaked at 61.3 ± 14.8 at the 24-month follow-up. This robust upward trajectory was highly significant across the designated time points (*p* < 0.001) (Figure 3B). Consistent with the PSQI findings, the longitudinal trajectories of HRQOL improvements were similar across both Responders and Non-Responders (Figure 3D). This further highlights that QoL enhancements occur alongside VNS therapy independent of the magnitude of the seizure-reduction benefit.

#### 3.2.4 GEE modeling

To thoroughly evaluate whether the observed improvements in sleep and QOL were statistically associated with device therapy over time while accounting for dynamic shifts in disease severity, a newly re-analyzed Generalized Estimating Equations (GEE) model was constructed. Rather than relying on a static, post-baseline 24-month responder classification, we incorporated the actual concurrent seizure frequency at each specific time point (6, 12, and 24 months) as a continuous, time-varying covariate. The model also adjusted for baseline psychiatric comorbidity (21, 22).

**Figure 4:**
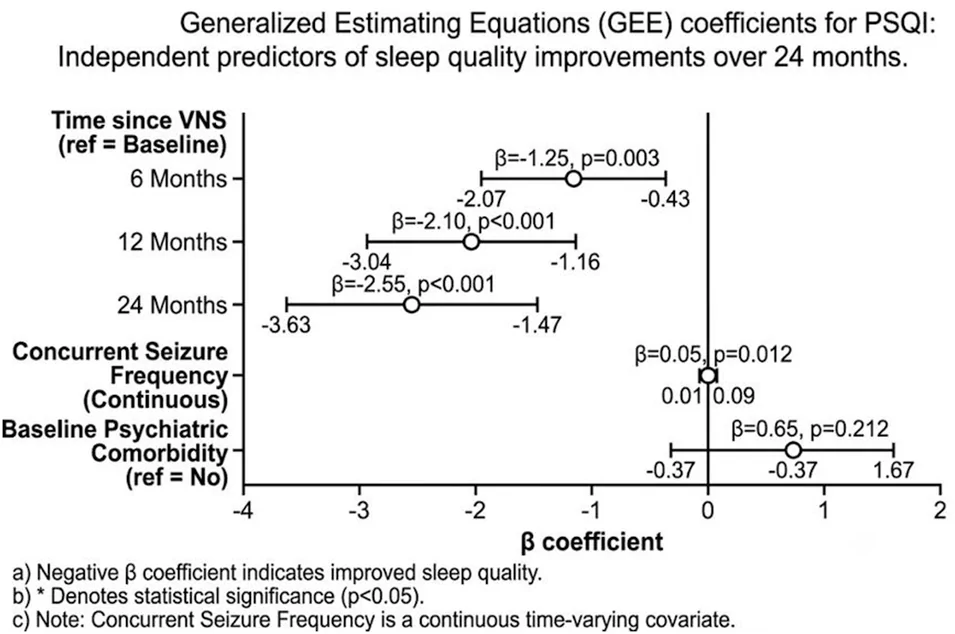
Generalized estimating equations (GEE) coefficients for PSQI: adjusted predictors of sleep quality improvements over 24 months. Forest plot illustrating adjusted longitudinal associations of sleep quality improvements over 24 months post-vagus nerve stimulation (VNS) implantation. After adjusting for concurrent seizure frequency as a continuous time-varying covariate and baseline psychiatric comorbidities (Hamilton Depression or Anxiety Rating Scale score ≥8), time post-implantation (6, 12, and 24 months) remained a highly significant predictor of PSQI improvement (*p* < 0.01 for all time points). A negative *β* coefficient for the PSQI indicates a reduction in the score, correlating with improved sleep quality. The plot also presents the effect sizes and 95% confidence intervals for the continuous time-varying covariate “concurrent seizure frequency” and baseline psychiatric comorbidity.

**Figure 5:**
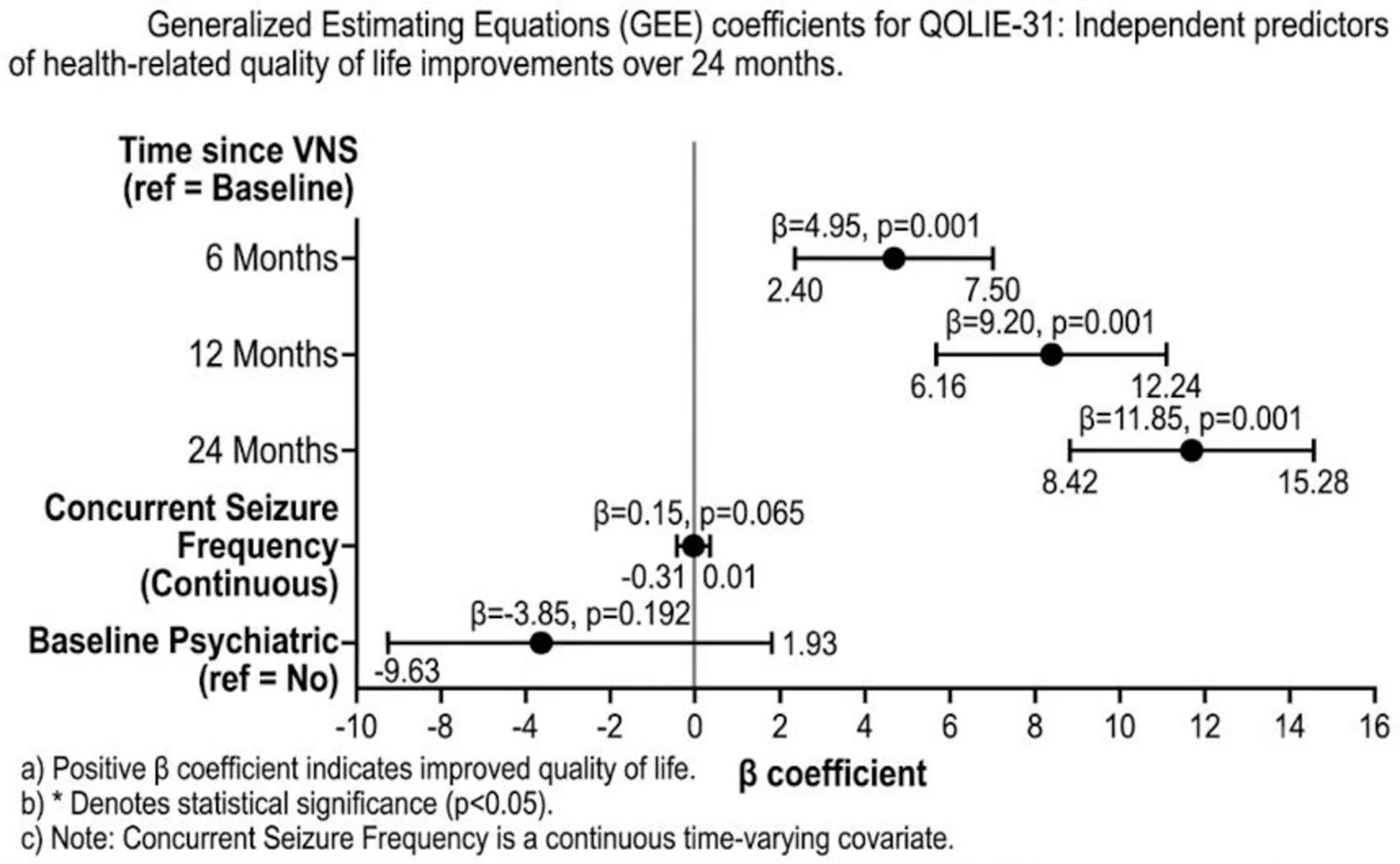
Generalized estimating equations (GEE) coefficients for QOLIE-31: adjusted predictors of health-related quality of life improvements over 24 months. Forest plot illustrating adjusted longitudinal associations of HRQOL improvements. Independent of the degree of seizure-frequency reduction (modeled as a continuous time-varying covariate) and the patient's baseline psychiatric comorbidity burden, time post-VNS implantation emerged as a highly significant adjusted predictor of increased QOLIE-31 scores (*p* < 0.001 for all time points). A positive *β* coefficient for the QOLIE-31 indicates an increase in the score, correlating with an improved quality of life. The plot additionally displays the statistical effects of the continuous covariate “concurrent seizure frequency” and baseline psychiatric comorbidity.

Crucially, the GEE analysis revealed that the Time factor remained a highly significant predictor for improvements in both PSQI (*p* < 0.01 for all time points) and QOLIE-31 (*p* < 0.001 for all time points), establishing a robust adjusted longitudinal association that persisted even after controlling for concurrent seizure frequency and baseline psychiatric comorbidities (Table 3; Figures 4, 5). These findings confirm that the multifaceted neurobehavioral and physiological benefits of VNS are sustained alongside therapy and are not exclusively dictated by the concurrent magnitude of seizure-frequency reduction in this cohort.

**Table 3:** Generalized estimating equations (GEE) modeling adjusted longitudinal associations with improvements in subjective sleep quality (PSQI) and quality of life (QOLIE-31).

Predictor Variables	*β* Coefficient	Standard Error (SE)	95% CI	*p*-value
Model 1: Factors associated with PSQI score				
**Time since VNS** *(ref = Baseline)*				
6 Months	−1.25	0.42	−2.07 to −0.43	0.003*
12 Months	−2.10	0.48	−3.04 to −1.16	<0.001*
24 Months	−2.55	0.55	−3.63 to −1.47	<0.001*
Concurrent Seizure Frequency (Continuous)	0.05	0.02	0.01 to 0.09	0.012
Baseline Psychiatric Comorbidity *(ref = No)*	0.65	0.52	−0.37 to 1.67	0.212
Model 2: Factors associated with QOLIE-31 score				
**Time since VNS** *(ref = Baseline)*				
6 Months	4.95	1.30	2.40 to 7.50	<0.001*
12 Months	9.20	1.55	6.16 to 12.24	<0.001*
24 Months	11.85	1.75	8.42 to 15.28	<0.001*
Concurrent Seizure Frequency (Continuous)	−0.15	0.08	−0.31 to 0.01	0.065
Baseline Psychiatric Comorbidity *(ref = No)*	−3.85	2.95	−9.63 to 1.93	0.192

CI, confidence interval; GEE, Generalized Estimating Equations; PSQI, Pittsburgh Sleep Quality Index; QOLIE-31, Quality of Life in Epilepsy-31; VNS, vagus nerve stimulation.

The asterisk (*) denotes statistical significance at *p* < 0.05.

## 4 Discussion

The present 24-month retrospective cohort study systematically evaluated the longitudinal trajectories of subjective sleep quality and health-related quality of life (HRQOL) in adult patients with drug-resistant epilepsy (DRE) undergoing vagus nerve stimulation (VNS). Our data demonstrate three principal findings. First, consistent with the established literature, VNS was associated with a significant and progressive reduction in monthly seizure frequency over the two-year observation period (4, 23, 24). Second, and more importantly, we observed profound, cumulative improvements in both the Pittsburgh Sleep Quality Index (PSQI) and the Quality of Life in Epilepsy-31 (QOLIE-31) scores across the entire cohort (24, 25). Third, by employing generalized estimating equations (GEE) that incorporated concurrent seizure frequency as a continuous, time-varying covariate, we found that the temporal gains in sleep quality and HRQOL exhibited a robust adjusted longitudinal association with VNS therapy, which remained statistically significant even after accounting for contemporaneous seizure-frequency reduction and baseline psychiatric comorbidity. These findings suggest that the neurobehavioral benefits of VNS are not exclusively tethered to the degree of macroscopic seizure control, although causality and complete physiological independence cannot be established in this observational design.

Our findings align with and extend the emerging literature on VNS-associated improvements in sleep and quality of life. Prior multicenter studies have demonstrated that VNS implantation is associated with significant improvements in both subjective sleep quality and health-related quality of life, as measured by PSQI and QOLIE-31 (26). Importantly, while some studies have found that improvements in depressive and anxiety symptoms were related to responder status, they did not consistently detect a significant association between PSQI/QOLIE-31 changes and seizure control (25). Pediatric and adult reviews have similarly noted that VNS improves cognitive function and sleep quality as a parallel effect associated with the control of epileptic seizures, yet these studies did not provide granular longitudinal data on the temporal evolution of these improvements (27).

Sleep dysfunction is highly prevalent among patients with DRE, driven by a complex interplay of nocturnal epileptiform activity, the neurodepressant side effects of polypharmacy, and underlying network hyperexcitability (28, 29). The baseline PSQI scores in our cohort (mean >8) confirmed substantial subjective sleep disturbances. Following VNS implantation, we observed a steady, time-dependent decline in PSQI scores, with significant changes detectable as early as six months and sustained through 24 months. Importantly, in our GEE model, the “time since implantation” factor retained a strong predictive value for PSQI improvement (*β* = –2.55 at 24 months, *p* < 0.001) independent of the concurrent seizure frequency entered as a continuous covariate. From a neuroanatomical perspective, this adjusted association is biologically plausible. Vagal afferent fibers project densely to the nucleus tractus solitarius (NTS), which in turn maintains direct excitatory projections to the locus coeruleus and dorsal raphe nucleus—key brainstem nuclei that regulate the ascending reticular activating system and sleep–wake transitions (30, 31). Chronic VNS may therefore stabilize these monoaminergic networks, promoting sleep consolidation through mechanisms that are at least partially distinct from those governing seizure threshold. Nonetheless, given that our primary outcome relied on the self-reported PSQI rather than polysomnography, we interpret these changes as improvements in subjective sleep quality rather than objectively verified modifications of sleep macrostructure (e.g., slow-wave or REM sleep) (32, 33).

A parallel upward trajectory was observed for overall HRQOL, measured by the QOLIE-31. Historically, the therapeutic efficacy of palliative epilepsy surgery has been predominantly gauged by the rigid 50% responder metric, implicitly assuming that psychosocial benefits are strictly contingent upon a substantial reduction in seizure counts. Our findings challenge this seizure-centric paradigm (1, 34). Although the concurrent seizure frequency showed a negative directionality with QOLIE-31 (*β* = –0.15), this association did not reach statistical significance (*p* = 0.065)—likely due to the limited sample size. Crucially, the GEE model demonstrated that the time-related increments in QOLIE-31 (*β* = 11.85 at 24 months, *p* < 0.001) were pronounced even among patients with less than 50% seizure reduction. This does not permit a claim of “independence” from seizure control; however, it does indicate that VNS may confer additional, durable HRQOL benefits that are not completely overshadowed by residual seizures. These benefits might be partially attributable to unmeasured improvements in seizure severity, post-ictal recovery duration, or daytime somnolence—parameters not captured by crude seizure frequency counts—or to direct limbic and cortical network modulation that enhances psychological resilience and subjective well-being (35).

Another important confounder in epilepsy outcome research is baseline mood disorder, as depression and anxiety heavily skew both sleep metrics and HRQOL inventories (36). Some earlier reports have suggested that the non-seizure benefits of VNS may primarily reflect its antidepressant properties (37, 38). To address this, we adjusted our GEE models for baseline psychiatric comorbidity defined by HAMD/HAMA scores ≥8. In our analysis, although baseline psychiatric burden exhibited a negative trend (*β* = –3.85 for QOLIE-31, *p* = 0.192), it did not significantly alter the robust time-dependent effects. This suggests that the observed improvements in sleep and HRQOL are unlikely to be mere epiphenomena of alleviating pre-existing depression, although future studies with larger samples and serial psychiatric assessments are needed to disentangle these inter-relationships fully.

We must acknowledge several important methodological limitations that temper the interpretation of our findings. First, the single-center, retrospective design inherently limits external generalizability (39), and the modest cohort size (*N* = 29) restricts statistical power for subgroup analyses—particularly regarding different epilepsy etiologies or specific antiseizure medication regimens. The absence of a control group (e.g., untreated, wait-list, or alternative-therapy arms) is a critical limitation. Without a comparator, the observed effects of time are inseparable from several unmeasured confounders, including natural fluctuation in seizure burden, adjustments in antiseizure or psychiatric medications, increased clinical contact and care coordination, and changes in family or psychosocial support over the 24-month period. Consequently, although we present adjusted longitudinal associations, we cannot attribute the benefits solely to a direct physiological effect of VNS. Second, we explicitly required patients to complete the 24-month follow-up for inclusion. While this ensures data completeness (all 29 patients provided data at all four time points), it inevitably introduces selection and survivorship bias. Our results may not reflect the outcomes of patients who withdrew earlier due to worsening seizures, severe adverse events, or lack of perceived efficacy, and our conclusions should not be extrapolated to those populations. Third, our assessment of sleep relied entirely on the PSQI, a validated subjective instrument. Future prospective investigations must integrate objective physiological metrics to empirically validate these subjective improvements.

Despite these limitations, this longitudinal analysis underscores the multifaceted therapeutic utility of VNS in the DRE treatment algorithm. The sustained, clinically meaningful improvements in subjective sleep quality and HRQOL—observed even after adjusting for concurrent seizure frequency—advocate for expanding the evaluation framework beyond simple seizure diaries. In clinical practice, physicians should counsel patients and families that the palliative value of VNS extends well beyond seizure counting, potentially offering meaningful neurobehavioral benefits even in those who do not achieve the traditional 50% responder threshold. To definitively establish the extent to which these benefits are physiologically dissociable from seizure control, future multicenter, prospective cohorts with objective sleep monitoring, comprehensive time-varying covariate collection, and a comparator group are urgently needed.

## 5 Conclusion

In conclusion, this 24-month retrospective cohort study demonstrates that VNS therapy is associated with significant, progressive, and sustained improvements in subjective sleep quality and overall health-related quality of life in adult patients with DRE. Moreover, our adjusted longitudinal analysis indicates that these neurobehavioral benefits are not exclusively dependent on the magnitude of concurrent seizure-frequency reduction. However, given the uncontrolled, single-center design and the lack of a comparator group, these findings should be interpreted as adjusted longitudinal associations rather than causal evidence of a seizure-independent physiological effect. Future prospective, controlled studies with objective sleep monitoring and comprehensive time-varying covariate collection are essential to confirm the multifaceted therapeutic utility of VNS in the holistic management of DRE.

## Data Availability

The original contributions presented in the study are included in the article/Supplementary Material, further inquiries can be directed to the corresponding author.
