# Supplementary material for: Longitudinal association of subjective sleep quality and health-related quality of life improvements with Vagus nerve stimulation in drug-resistant epilepsy: a 24-month retrospective cohort study
Source: Front Surg. 2026 Aug 18;13:1914334. doi: 10.3389/fsurg.2026.1914334 (PMC13529677; doi:10.3389/fsurg.2026.1914334)
Supplement: Supplementary file 1 [file Table1.docx]

**Supplementary Table 1. VNS Stimulation Parameters and Clinical Characteristics Across Follow-Up Timepoints (N = 29)**

| **Pt.** | Sex | **VNS Device Model** | **6-Month Parameters (Output / PW / Freq / Duty)** | **12-Month Parameters (Output / PW / Freq / Duty)** | **24-Month Parameters (Output / PW / Freq / Duty)** | **AutoStim / Magnet Mode** | **24-Mo Outcome (Reduction %)** |
| --- | --- | --- | --- | --- | --- | --- | --- |
| **P01** | F | Pins G112 | 1.25 / 250 / 20 / 30s-5m | 1.50 / 250 / 20 / 30s-5m | 1.75 / 250 / 20 / 30s-3m | Auto 2.00 / Mag 2.00 | Non-responder |
| **P02** | F | Pins G112 | 1.50 / 250 / 30 / 30s-5m | 1.75 / 250 / 20 / 30s-5m | 2.00 / 250 / 20 / 30s-5m | Auto 2.00 / Mag 2.25 | Non-responder |
| **P03** | F | Pins G112 | 1.25 / 250 / 20 / 30s-5m | 1.50 / 250 / 20 / 30s-5m | 1.50 / 250 / 20 / 30s-5m | Auto 1.75 / Mag 1.75 | Non-responder |
| **P04** | F | Pins G112 | 1.50 / 500 / 20 / 30s-5m | 1.75 / 250 / 20 / 30s-5m | 2.25 / 250 / 20 / 30s-3m | N/A / Mag 2.50 | Non-responder |
| **P05** | F | Pins G112 | 1.75 / 250 / 20 / 30s-5m | 2.00 / 250 / 20 / 30s-3m | 2.25 / 250 / 20 / 7s-14s | Auto 2.50 / Mag 2.50 | Non-responder |
| **P06** | F | Pins G112 | 1.25 / 250 / 20 / 30s-5m | 1.50 / 250 / 20 / 30s-5m | 1.75 / 250 / 20 / 30s-5m | Auto 2.00 / Mag 2.00 | Responder |
| **P07** | F | Pins G112 | 1.00 / 250 / 20 / 30s-5m | 1.25 / 250 / 20 / 30s-5m | 1.50 / 250 / 20 / 30s-5m | Auto 1.75 / Mag 1.75 | Non-responder |
| **P08** | M | Pins G112 | 1.50 / 250 / 30 / 30s-5m | 1.75 / 250 / 20 / 30s-5m | 2.00 / 250 / 20 / 30s-5m | N/A / Mag 2.25 | Non-responder |
| **P09** | F | Pins G112 | 1.50 / 250 / 20 / 30s-5m | 1.75 / 250 / 20 / 30s-3m | 2.00 / 250 / 20 / 30s-3m | Auto 2.25 / Mag 2.25 | Seizure Free |
| **P10** | M | Pins G112 | 1.25 / 250 / 20 / 30s-5m | 1.50 / 250 / 20 / 30s-5m | 1.75 / 250 / 20 / 30s-5m | Auto 2.00 / Mag 2.00 | Non-responder |
| **P11** | F | Pins G112 | 1.25 / 250 / 20 / 30s-5m | 1.50 / 250 / 20 / 30s-5m | 1.75 / 250 / 20 / 30s-3m | Auto 2.00 / Mag 2.00 | Responder |
| **P12** | M | Pins G112 | 1.75 / 250 / 20 / 30s-3m | 2.00 / 250 / 20 / 30s-3m | 2.25 / 250 / 20 / 30s-1.8m | Auto 2.50 / Mag 2.50 | Non-responder |
| **P13** | F | Pins G112 | 1.25 / 250 / 20 / 30s-5m | 1.50 / 250 / 20 / 30s-5m | 1.75 / 250 / 20 / 30s-5m | N/A / Mag 2.00 | Responder |
| **P14** | M | Pins G112 | 1.50 / 250 / 20 / 30s-5m | 1.75 / 250 / 20 / 30s-5m | 2.00 / 250 / 20 / 30s-3m | Auto 2.25 / Mag 2.25 | Responder |
| **P15** | F | Pins G112 | 1.25 / 250 / 20 / 30s-5m | 1.50 / 250 / 20 / 30s-5m | 1.50 / 250 / 20 / 30s-5m | Auto 1.75 / Mag 1.75 | Responder |
| **P16** | M | Pins G112 | 1.50 / 250 / 20 / 30s-5m | 1.75 / 250 / 20 / 30s-3m | 2.00 / 250 / 20 / 30s-3m | Auto 2.25 / Mag 2.25 | Responder |
| **P17** | F | Pins G112 | 1.00 / 500 / 20 / 30s-5m | 1.25 / 250 / 20 / 30s-5m | 1.50 / 250 / 20 / 30s-5m | Auto 1.75 / Mag 1.75 | Non-responder |
| **P18** | M | Pins G112 | 1.75 / 250 / 20 / 30s-5m | 2.00 / 250 / 20 / 30s-3m | 2.25 / 250 / 20 / 30s-3m | Auto 2.50 / Mag 2.50 | Responder |
| **P19** | F | Pins G112 | 1.25 / 250 / 20 / 30s-5m | 1.50 / 250 / 20 / 30s-5m | 1.75 / 250 / 20 / 30s-5m | Auto 2.00 / Mag 2.00 | Responder |
| **P20** | M | Pins G112 | 1.50 / 250 / 20 / 30s-5m | 1.75 / 250 / 20 / 30s-5m | 2.00 / 250 / 20 / 30s-3m | Auto 2.25 / Mag 2.25 | Responder |
| **P21** | M | Pins G112 | 1.25 / 250 / 20 / 30s-5m | 1.50 / 250 / 20 / 30s-5m | 1.75 / 250 / 20 / 30s-5m | N/A / Mag 2.00 | Responder |
| **P22** | F | Pins G112 | 1.50 / 250 / 20 / 30s-5m | 1.75 / 250 / 20 / 30s-3m | 2.00 / 250 / 20 / 30s-3m | Auto 2.25 / Mag 2.25 | Responder |
| **P23** | M | Pins G112 | 1.25 / 250 / 20 / 30s-5m | 1.50 / 250 / 20 / 30s-5m | 1.50 / 250 / 20 / 30s-5m | Auto 1.75 / Mag 1.75 | Non-responder |
| **P24** | F | Pins G112 | 1.50 / 250 / 20 / 30s-5m | 1.75 / 250 / 20 / 30s-5m | 2.00 / 250 / 20 / 30s-3m | Auto 2.25 / Mag 2.25 | Responder |
| **P25** | M | Pins G112 | 1.25 / 250 / 20 / 30s-5m | 1.50 / 250 / 20 / 30s-5m | 1.75 / 250 / 20 / 30s-5m | Auto 2.00 / Mag 2.00 | Responder |
| **P26** | F | Pins G112 | 1.00 / 250 / 20 / 30s-5m | 1.25 / 250 / 20 / 30s-5m | 1.50 / 250 / 20 / 30s-5m | N/A / Mag 1.75 | Non-responder |
| **P27** | M | Pins G112 | 1.50 / 250 / 20 / 30s-5m | 1.75 / 250 / 20 / 30s-3m | 2.00 / 250 / 20 / 30s-3m | Auto 2.25 / Mag 2.25 | Responder |
| **P28** | F | Pins G112 | 1.25 / 250 / 20 / 30s-5m | 1.50 / 250 / 20 / 30s-5m | 1.75 / 250 / 20 / 30s-5m | Auto 2.00 / Mag 2.00 | Responder |
| **P29** | M | Pins G112 | 1.75 / 250 / 20 / 30s-5m | 2.00 / 250 / 20 / 30s-3m | 2.25 / 250 / 20 / 30s-1.8m | Auto 2.50 / Mag 2.50 | Responder |

**Abbreviations:**

VNS, Vagus Nerve Stimulation; PW, Pulse Width; Freq, Frequency; Duty, Duty Cycle (ON time / OFF time); AutoStim, heart rate-based automatic stimulation mode; N/A, Not Applicable (device model without AutoStim feature).

**Responder Definition:**

Patients with a ≥ 50% reduction in mean monthly seizure frequency compared to baseline at 24 months were classified as Responders.

**Summary Statistics:**

6-Month Mean Output Current: 1.39 ± 0.22 mA; 12-Month Mean Output Current: 1.62 ± 0.22 mA; 24-Month Mean Output Current: 1.87 ± 0.25 mA.

Overall Responder Rate at 24 Months (≥ 50% reduction): 58.6% (17/29).
